# Mactinin, a fragment of cytoskeletal α-actinin, is a novel inducer of heat shock protein (Hsp)-90 mediated monocyte activation

**DOI:** 10.1186/1471-2121-10-60

**Published:** 2009-08-28

**Authors:** Sharon D Luikart, Angela Panoskaltsis-Mortari, Timothy Hinkel, Robert T Perri, Kalpna Gupta, Theodore R Oegema, Pankaj Gupta

**Affiliations:** 1 Hematology/Oncology Section (111E), Veterans Affairs Medical Center, Minneapolis, MN 55417, USA; 2 Department of Medicine, University of Minnesota, Minneapolis, Minnesota, 55455, USA; 3 Department of Pediatrics, University of Minnesota, Minneapolis, Minnesota, 55455, USA; 4 Department of Biochemistry, Rush University, Chicago, Illinois, 60612, USA

## Abstract

**Background:**

Monocytes, their progeny such as dendritic cells and osteoclasts and products including tumor necrosis factor (TNF)-α, interleukin (IL)-1α and IL-1β play important roles in cancer, inflammation, immune response and atherosclerosis. We previously showed that mactinin, a degradative fragment of the cytoskeletal protein α-actinin, is present at sites of monocytic activation in vivo, has chemotactic activity for monocytes and promotes monocyte/macrophage maturation. We therefore sought to determine the mechanism by which mactinin stimulates monocytes.

**Results:**

Radiolabeled mactinin bound to a heterocomplex on monocytes comprised of at least 3 proteins of molecular weight 88 kD, 79 kD and 68 kD. Affinity purification, mass spectroscopy and Western immunoblotting identified heat shock protein (Hsp)-90 as the 88 kD component of this complex. Hsp90 was responsible for mediating the functional effects of mactinin on monocytes, since Hsp90 inhibitors (geldanamycin and its analogues 17-allylamino-17-demethoxygeldanamycin [17-AAG] and 17-(dimethylaminoethylamino)-17-demethoxygeldanamycin [17-DMAG]) almost completely abrogated the stimulatory activity of mactinin on monocytes (production of the pro-inflammatory cytokines IL-1α, IL-1β and TNF-α, as well as monocyte chemotaxis).

**Conclusion:**

Mactinin is a novel inducer of Hsp90 activity on monocytes and may serve to perpetuate and augment monocytic activation, thereby functioning as a "matrikine." Blockage of this function of mactinin may be useful in diseases where monocyte/macrophage activation and/or Hsp90 activity are detrimental.

## Background

Cell migration and chemotaxis that occur in malignancies and inflammatory processes may deposit the focal adhesion component α-actinin in their migratory path [1]. We previously showed that extracellular α-actinin is degraded by monocyte-secreted urokinase to generate a specific fragment (which we named mactinin) [2]. Mactinin is found at various sites of monocytic activation in vivo [2-4], has chemotactic activity for monocytes [4] and promotes monocyte/macrophage maturation [5]. These findings suggest that mactinin is a functionally important mediator of monocytic activity.

Monocytes and macrophages play pivotal roles during inflammatory and immune processes by releasing various cytokines including tumor necrosis factor (TNF)-α, interleukin (IL)-1α and IL-1β, chemokines, enzymes and other factors [6]. In some disease processes such as infections [6] and wound healing [3,7,8], macrophage activity may be beneficial in promoting healing. In other diseases, such as arthritis [9-13] and atherosclerosis [14,15], macrophage activation may contribute to pathogenesis and propagation. The monocyte/macrophage system also plays an integral role in malignancies by secretion of these cytokines, generation of dendritic cells and osteoclasts and modulation of the immune response [reviewed in [16,17]].

In the current study, we examined the mechanism mediating the stimulatory effect of mactinin on monocytes. We show here that mactinin binds to a heterocomplex including heat shock protein (Hsp)-90 on monocytes, and that Hsp90 is critically important for the stimulatory activity of mactinin on monocytes since inhibition of Hsp90 almost completely blocked mactinin-induced cytokine production and migration of monocytes. Hsp90 is a molecular chaperone whose activity promotes chemotaxis, migration, proliferation and cytokine secretion in malignant and endothelial cells and in monocytes [18-28]. Our identification of mactinin as a novel inducer of Hsp90 activity on monocytes therefore has important implications for diverse conditions including malignancies, autoimmune disease, inflammation and atherosclerosis.

## Results

### Mactinin stimulates IL-1α, IL-1β and TNF-α production by monocytes

**Figure 1:**
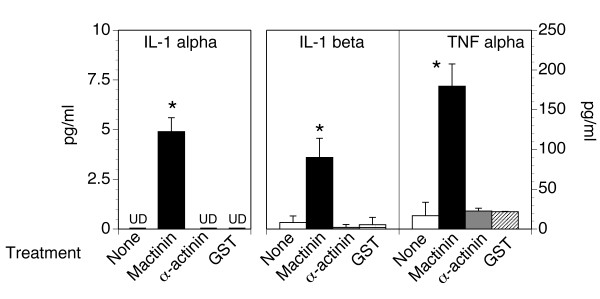
**Mactinin stimulates production of cytokines from monocytes**. Human peripheral blood monocytes were incubated for 24 hrs with 100 nM mactinin, 100 nM α-actinin, 10 nM glutathione-S-transferase (GST), or no treatment. The concentrations of the indicated cytokines were determined in the supernatant. UD: undetectable at an assay sensitivity of 1.0 pg/ml. Data is shown as the mean +/- SEM. N = 3–4. Significance of differences between no treatment and mactinin: *P < 0.01.

Peripheral blood monocytes were isolated and cultured for 24 h with 100 nM mactinin, 100 nM α-actinin, 10 nM GST or medium alone (no treatment). The GST condition was included in order to control for the 10% contaminating GST in our mactinin preparation. Supernatants were recovered and centrifuged to remove nonadherent cells and aliquots assayed for the 3 cytokines. As shown in Fig. 1, the levels of IL-1α, IL-1β, and TNFα were significantly increased in the supernatants of mactinin-treated monocytes. Control cultures treated with α-actinin or GST did not show any increase in cytokine production. Mactinin did not stimulate the production of granulocyte macrophage colony-stimulating factor (GM-CSF), interferon (IFN)-α, IL-12, macrophage colony-stimulating factor (M-CSF), or macrophage inhibitory protein (MIP)-1α (not shown). These findings indicate that mactinin directly stimulates the production of specific pro-inflammatory cytokines from monocytes.

### Mactinin binds to monocytes

**Figure 2:**
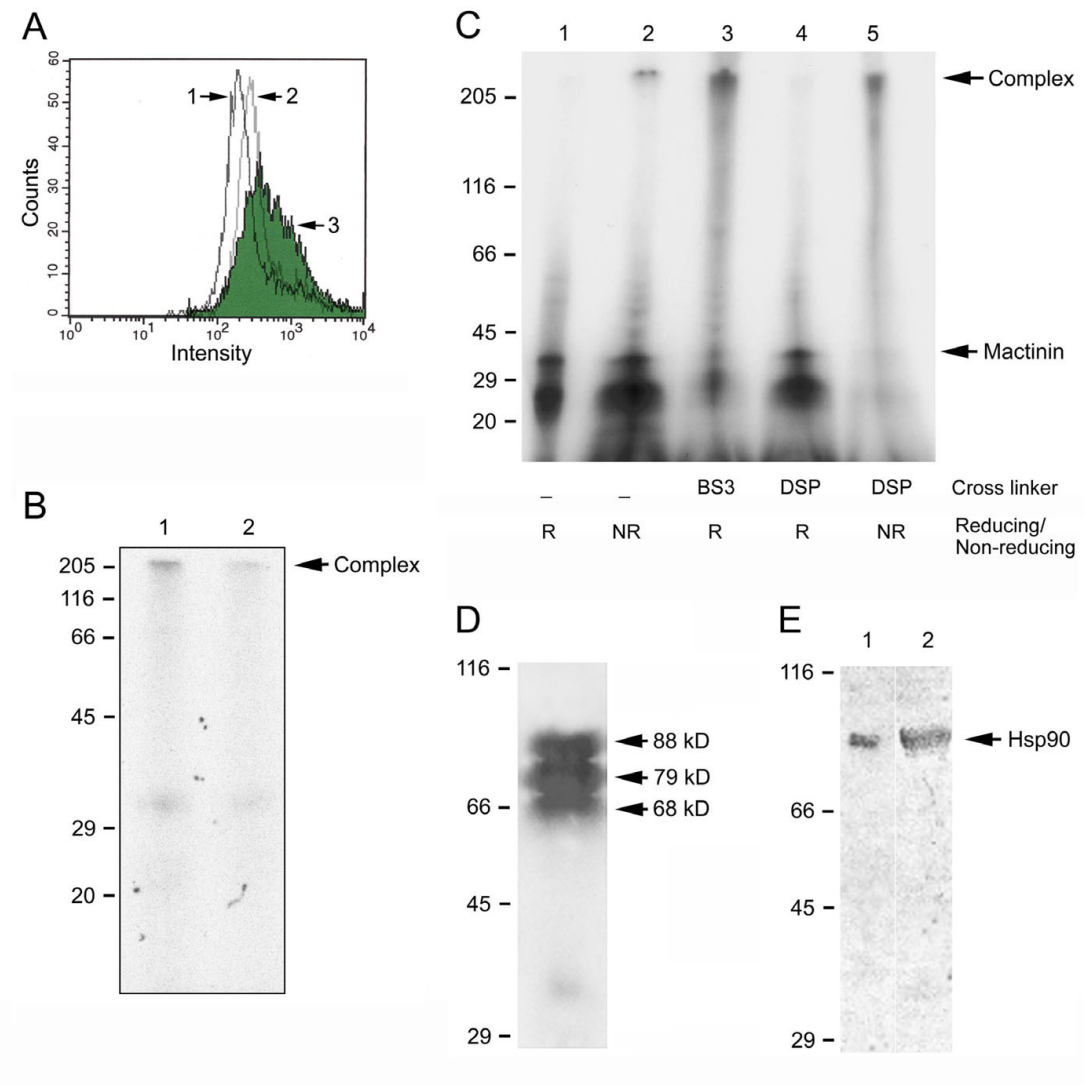
**Mactinin binds to a Hsp90 containing complex on the surface of monocytoid cells**. **A: Mactinin binds directly to peripheral blood monocytes**. Monocytes incubated without (plot 1) or with (plot 3: shaded area) 100 nM mactinin were analyzed by flow cytometry to detect mactinin bound to the cell surface. The control condition (cells incubated with mactinin and stained with isotype matched IgG_1 _antiserum) is shown in plot 2. One of four representative experiments is shown. **B: Mactinin binds specifically in a high molecular weight complex on monocytoid HL-60 cells**. HL-60 cells were pre-incubated with or without excess unlabeled (cold) mactinin, and then with [^125^I]mactinin. Cell lysate was resolved by 12% SDS-PAGE under non-reducing conditions, and [^125^I]mactinin detected using autoradiography. Lane 1: high molecular weight complex on cells incubated with [^125^I]mactinin alone. Lane 2: marked reduction in bound [^125^I]mactinin in cells pre-incubated with unlabeled mactinin. **C: Mactinin binds to monocytoid HL-60 cells**. HL-60 cells were biotinylated and then incubated for 1 h at 4°C with 100 nM [^125^I]-mactinin, washed, and incubated without or with the cross-linkers DSP or BS^3^, lysed, and resolved by 6–20% gradient SDS-PAGE under non-reducing or reducing conditions. Lane 1: 31 kD radiolabeled mactinin alone, reducing conditions (no cells). Lane 2: radiolabeled mactinin bound to a high molecular weight complex on HL-60 cells, non-reducing conditions, no cross-linker. Lane 3: retention of most of the radiolabeled mactinin in the complex in presence of the impermeable, non-cleavable cross-linker BS^3^, reducing conditions. Lane 4: 31 kD radiolabeled mactinin separated from the binding complex under reducing conditions, cleavable cross-linker DSP. Lane 5: retention of nearly all of the radiolabeled mactinin in the complex in presence of the cross-linker DSP, non-reducing conditions. **D: Mactinin binds to 3 distinct proteins on HL-60 cells**. HL-60 cells were biotinylated (to detect cell surface proteins that bind to mactinin) and incubated with 100 nM mactinin. The HL-60 cell protein-mactinin complex (the material in lane 5 in Fig. 2C) was electroeluted, reduced, run over a mactinin column, and the bound proteins were resolved by 6–20% gradient SDS-PAGE. Biotinylated HL-60 proteins (88, 79 and 68 kD) that bound to the mactinin affinity column were detected by Western immunoblotting. **E: Identification of Hsp90 in the mactinin-binding complex**. The membrane shown in Fig. 2D was stripped and re-probed using an anti-Hsp90 antibody. A single 90 kD band was detected (lane 1). Recombinant Hsp90 was run in lane 2.

To assess whether mactinin binds to peripheral blood monocytes, these cells were incubated with or without mactinin and then stained with antiserum to mactinin or isotype matched (IgG_1_) control pre-immune antiserum. Bound mactinin was measured using flow cytometry (Fig. 2A). There was a significantly higher percentage of positively staining monocytes that had been incubated with mactinin (44 ± 2%; Fig 2A plot 3) than monocytes not incubated with mactinin (15 ± 1%; p = 0.003; Fig 2A plot 1) or monocytes incubated with mactinin but detected with control IgG_1 _antiserum (22% ± 1%; p = 0.005; Fig 2A plot 2). Furthermore, the mean fluorescence intensity of monocytes incubated with mactinin (509 ± 2%) was significantly higher than that of monocytes incubated without mactinin (265 ± 1%; p < 0.0001) or monocytes incubated with mactinin but detected with control IgG_1 _antiserum (388 ± 4; p < 0.002). We saw a similar increase in positive staining and mean fluorescence in HL-60 cells incubated with mactinin (data not shown). Thus, mactinin binds to both monocytes and monocytoid cells.

### Mactinin binds to a specific high molecular weight complex on HL-60 cells

We next examined the specificity of mactinin binding to HL-60 cells, by determining the ability of excess unlabeled (cold) mactinin to compete for the binding of [^125^I]mactinin to HL-60 cells. HL-60 cells were pre-incubated with or without unlabeled mactinin, followed by addition of [^125^I]mactinin. Autoradiography of the clarified lysates resolved by 12% non-reducing SDS-PAGE detected a high molecular weight complex on cells that were incubated with [^125^I]mactinin (Fig. 2B, lane 1). This complex was markedly reduced in cells pre-incubated with unlabeled mactinin (Fig. 2B, lane 2). These results suggest that mactinin can compete for a specific and potentially saturable binding site on HL-60 cells.

### Mactinin binds a hetero-complex of HL-60 cell membrane proteins including Hsp90

Mactinin has *in vitro *maturation promoting effects on both monocytes and HL-60 leukemia cells [5]. We therefore elected to use the plentiful HL-60 cell line for biochemical analysis of mactinin binding proteins. Biotin surface labeled HL-60 cells were incubated with 100 nM [^125^I]mactinin in the presence or absence of the cleavable, cell-permeable cross-linker DSP or the non-cleavable, cell-impermeable cross-linker BS^3^. The cells were lysed, centrifuged and proteins in the supernatant resolved by 6–20% gradient SDS-PAGE under reducing or non-reducing conditions. Autoradiography detected the 31 kD [^125^I]mactinin that was bound to HL-60 cells in the absence of a cross-linker (Fig. 2C, lane 2). Use of the cross-linker DSP demonstrated that most of the [^125^I]mactinin bound to a high molecular weight complex on HL-60 cells (Fig. 2C, lane 5). Under reducing conditions, the free [^125^I]mactinin could be separated out completely from the HL-60 protein complex seen with DSP (Fig. 2C, lane 4). Since BS^3 ^is not cleavable, the complex remained unchanged (with bound [^125^I]mactinin) under reducing conditions with BS^3 ^(Fig. 2C, lane 3).

To identify HL-60 proteins in the binding complex, the radiolabeled complex (from Fig. 2C, lane 5) was cut, electroeluted, dialyzed, reduced with DTT, run on a column of mactinin covalently bound to an Affi-Gel 15 matrix and eluted to select for mactinin-binding proteins. A purification step was necessary since mass spectrometry analysis of the resulting proteins from the unpurified complex showed many co-migrating proteins including myeloperoxidase, glucose-regulated protein 94, 78 kD glucose-regulated protein, calnexin, calreticulin precursor, moesin, Hsc70-ps1, protein disulfide isomerase-associated 4, prolyl 4-hydroxylase beta subunit and protein disulfide isomerase-associated 3 precursor. Nevertheless, even in the unpurified complex, more peptides matched Hsp90 (27% of all the peptides) than any other single protein.

The biotin-labeled HL-60 proteins that were eluted from matrix-bound mactinin were separated by SDS-PAGE and transferred to a PVDF membrane for detection of the proteins by Western immunoblotting for biotin. As shown in Fig. 2D, three protein bands of molecular weight 88, 79 and 68 kD were detected. A companion gel lane stained with Coomassie-blue visualized only the 88 kD band (not shown).

The identity of the 88 kD protein was confirmed by Western immunoblotting. Re-probing the membrane shown in Fig. 2D with antiserum to Hsp90 detected a single band of 88 kD (Fig. 2E, lane 1), as well as recombinant Hsp90 run in a separate lane as a positive control (Fig. 2E, lane 2). Together, the above data indicate that mactinin binds to a high molecular weight complex of at least 3 proteins on monocytoid HL-60 cells, and one of these proteins is Hsp90.

### Hsp90 mediates the stimulation of monocytes by mactinin

To determine if Hsp90 identified in the mactinin-binding complex is functionally involved in the activity of mactinin on monocytes, we assessed the effect of 3 separate Hsp90 inhibitors on mactinin-induced monocyte migration [4] and cytokine production.

**Figure 3:**
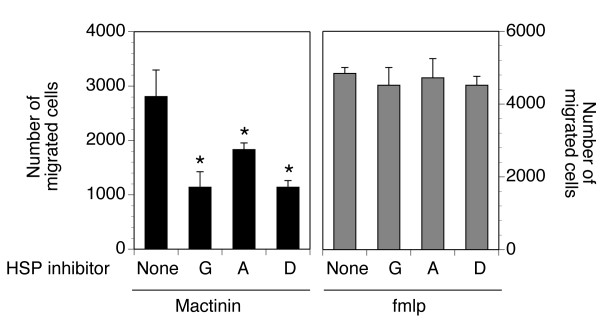
**Mactinin-induced monocyte migration is blocked by Hsp90 Inhibitors**. Monocytes were incubated with medium alone or with one of 3 Hsp90 inhibitors (G: geldanamycin; A: 17-AAG; D: 17-DMAG), prior to testing for mactinin-induced or fmpl-induced (control) chemotaxis. In the absence of mactinin or fmlp, 1302 +/- 122 cells migrated (not shown). Mactinin-induced migration was significantly inhibited by each of the Hsp90 inhibitors, whereas fmlp-induced migration was not. Data is shown as the mean +/- SEM. N = 3. Significance of differences in migration in the presence vs absence of Hsp90 inhibitors: *P < 0.01.

As shown in Fig. 3, all three Hsp90 inhibitors (geldanamycin, 17-allylamino-17-demethoxygeldanamycin [17-AAG] and 17-(dimethylaminoethylamino)-17-demethoxygeldanamycin [17-DMAG], completely suppressed monocyte chemotaxis, since the number of cells that migrated towards mactinin in the presence of the Hsp90 inhibitors was comparable to the number of cells that migrated in medium alone (without mactinin; negative control). In contrast, fmlp-induced chemotaxis was not inhibited. The concentrations of the Hsp90 inhibitors tested included 100, 200, and 400 nM geldanamycin, 0.5 and 1 μM 17-AAG, and 100 nM 17-DMAG, based on previous reports of *in vitro *Hsp90 inhibition studies [29,30]. All concentrations significantly inhibited migration without meaningful differences (not shown).

**Figure 4:**
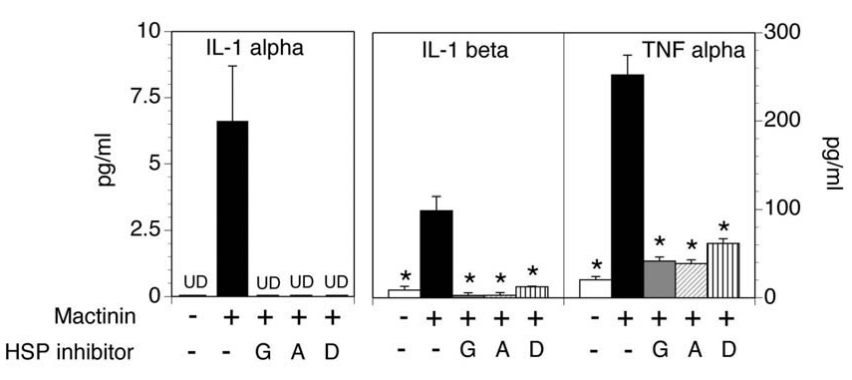
**Mactinin-induced production of cytokines from monocyts is blocked by Hsp90 inhibitors**. Monocytes were incubated for 24 hrs with medium alone, mactinin alone or mactinin + one of 3 Hsp90 inhibitors (G: geldanamycin; A: 17-AAG; D: 17-DMAG). The concentrations of the indicated cytokines were determined in the supernatant. UD: undetectable at an assay sensitivity of 1.0 pg/ml. Data is shown as the mean +/- SEM. N = 3–4. Significance of differences between mactinin alone and the other conditions: *P < 0.01.

Finally, we examined if the same Hsp90 inhibitors block mactinin-induced inflammatory cytokine production from monocytes (Fig. 4). Each of the three Hsp90 inhibitors completely blocked the production of IL-1α and IL-1β from monocytes. In contrast, production of TNF-α was not completely blocked since TNF-α levels in the presence of the Hsp90 inhibitors were higher than in medium alone, though it was markedly reduced compared to the levels seen with mactinin stimulation.

## Discussion

This study shows that mactinin induces Hsp90-mediated monocyte activation resulting in stimulation of cytokine secretion and migration. The recognition that degradative products of cytoskeletal proteins may influence Hsp90 activity in target cells has important implications for diverse conditions including malignancies, autoimmune disease, inflammation and atherosclerosis. Our findings also extend the current understanding of "matrikines," proteolytically released extracellular matrix-derived bioactive peptides that influence many types of cells and processes (reviewed in [31-33]).

Hsp90 is a highly conserved member of the group of molecular chaperones, is usually cytosolic [34,35] but may also be associated with lipid rafts in the cell membrane [36,37]. Hsps are constitutively expressed but enhanced in response to cellular stresses [38]. Their major function appears to be to act as protein chaperones, facilitating folding, oligomeric assembly, transport to a particular subcellular compartment, or switching between active and inactive protein conformation [39]. Hsp90 regulates many client proteins involved in signal transduction [40]. Other important functions include stimulation of chemotaxis, migration, proliferation and cytokine secretion in malignant and endothelial cells [18-22].

We show for the first time that Hsp90 is a functionally critical component of the mactinin-binding heterocomplex on monocytic cells. Two other proteins of molecular weight 68 and 79 kD found in this complex may possibly represent other Hsps, since previous studies have shown that Hsp90/70 forms a receptor complex in monocytes and macrophages [36,41]. Many Hsps interact with components of the actin cytoskeleton, perhaps modulating the cytoskeletal response to stress [42]. It has been reported that Hsp90 and Hsp100 bind actin [43], while the smaller Hsp20 has been reported to associate with both actin and α-actinin [44]. However, it is likely that the direct binding of extracellular mactinin is to other (as yet unidentified) protein(s) on the surface of HL-60 cells, which then form a hetero-complex with Hsp90.

In monocytes/macrophages and related cells, Hsp90 potently induces TNF-α and IL-1β expression and activation [23-26] and cell cycling and differentiation [27]. The Hsp-activated macrophage axis may also play an important role in atherosclerosis [reviewed in [28]]. Inhibitors of Hsp90, such as geldanamycin and its analogues, have been used to identify Hsp90 substrates [40,45]. We found that geldanamycin and two of its analogues markedly inhibited mactinin-induced chemotaxis and cytokine production. Their ability to inhibit chemotaxis was specific for mactinin since they had no significant effect on fmlp-induced chemotaxis. We also found that Hsp90 inhibitors blocked mactinin-induced cytokine production from monocytes, confirming that Hsp90 mediates the stimulatory activity of mactinin on monocytes.

Our results suggest that a cytoskeletal protein derivative (mactinin) may propagate inflammation, in part, by stimulating production of the pro-inflammatory cytokines IL-1α, IL-1β and TNF-α. These two cytokines are elevated in the serum and synovial fluid of patients with rheumatoid arthritis, and both are capable of inducing joint damage in experimental arthritis models [10]. The clinical efficacy of agents that block these cytokines in ameliorating bone and joint destruction in arthritis is now widely accepted [11].

Hsp90 induces urokinase-type plasminogen activator secretion [20] and urokinase secreted by activated monocytes degrades α-actinin to generate mactinin [2]. It is therefore possible that the binding and stimulation of Hsp90 activity on monocytes by mactinin itself (shown in the current study) may represent a positive feed-forward loop that further amplifies the immune/inflammatory response. We therefore speculate that agents that block mactinin may provide a novel strategy for inhibiting Hsp90 activity in diverse diseases.

## Conclusion

We have identified that Hsp90 mediates the stimulation of monocytes by mactinin, a fragment of the cytoskeletal protein α-actinin. Mactinin represents a novel inducer of Hsp90 activity on monocytes. Our findings suggest that inhibition of mactinin may be of benefit in diseases propagated by monocyte/macrophage activation or Hsp90 activity.

## Methods

### Isolation of peripheral blood monocytes

Mononuclear cells were isolated from blood samples obtained from healthy adult donors by density gradient centrifugation over Histopaque 1077 (Sigma, St. Louis, MO) as previously described [4]. Contaminating red cells were lysed in distilled water and monocytes were isolated using an LS separation column with a magnetic monocyte isolation kit in accordance with the manufacturer's instructions (Miltenyi Biotec, Auburn, CA). This negative selection method yielded more than 90% monocytes, as determined by CD14 expression, within three hours of phlebotomy. These studies were approved by the Institutional Review Board of the Minneapolis Veterans Affairs Medical Center.

### Cell Culture

HL-60 myeloid leukemia cells were purchased from the American Type Culture Collection (Manassas, VA) and maintained in RPMI medium with 50 μg/ml gentamicin and 15% fetal calf serum at 37°C and 5% CO_2_. We have previously reported that mactinin promotes monocytic maturation in this cell line [5].

### Source of mactinin

Dr. D.R. Critchley of the University of Leicester, U.K. kindly provided a PGEX2 vector which encodes the actin-binding domain, residues 2–269 of chicken smooth muscle α-actinin, fused with the carboxy terminus of glutathione S-transferase (GST) and containing an engineered thrombin cleavage site [2]. Fusion protein was expressed in *Escherichia coli*, and purified by affinity chromatography on immobilized glutathione. The fusion protein was then cleaved with thrombin (Calbiochem, San Diego, CA) to produce the actin-binding domain of α-actinin and the GST carrier. These products were then separated by reverse-phase high-performance liquid chromatography (HPLC) on a C-4 column. SDS-PAGE demonstrated that the mactinin was more than 90% of the total protein of pooled fractions, with the remaining 10% being GST. This 10–20 μM mactinin preparation from the C-4 column assayed negative for endotoxin with a Pyrotell chromogenic assay kit, which can detect 0.25 endotoxin units/ml (Associates of Cape Cod, Woods Hole, MA). Mactinin from the C-4 column was maintained in 30% acetonitrile and diluted in fresh, pyrogen-free water or tissue culture media for use in the various assays.

### Mactinin Antiserum

Purified recombinant chicken mactinin was modified by coupling with dinitrophenol and injected into two New Zealand white rabbits along with complete Freund's adjuvant, as described previously [2], to generate highly sensitive mactinin antiserum. Boosts were performed with peptide and incomplete Freund's adjuvant. These animal studies were approved by the Institutional Animal Care and Use Committee (IACUC). Antisera were screened, and sera reacting to mactinin were immunoaffinity-purified over a column of recombinant mactinin covalently bound to an Affi-Gel 15 matrix (Bio-Rad, Hercules, CA).

### Measurement of cytokine production by monocytes

Cytokine levels in culture supernatants were determined at the Cytokine Reference Lab (University of Minnesota) by multiplex assay using the Luminex system (Austin, TX) and human cytokine-specific bead sets (R&D Systems, Minneapolis, MN) or ELISA kits (Bender Medsystems, Burlingame, CA). Each set of experiments was repeated two to three times.

### Detection of binding of mactinin to monocytes using flow cytometry

Two million monocytes were incubated with or without 100 nM recombinant mactinin in PBS containing 0.5% bovine serum for 90 min at 4°C with occasional mixing. Monocytes were then washed and incubated with no antiserum, control pre-immune rabbit IgG_1 _or affinity-purified rabbit antiserum against recombinant mactinin for 30 minutes at 4°C with occasional mixing, followed by another wash and incubation with goat anti-rabbit IgG-FITC (Sigma) for 30 min at 4°C in the dark with occasional mixing. Stained cells were washed and analyzed using a flow cytometer (Beckton Dickinson Model 450).

### Radiolabeling of mactinin

Recombinant mactinin is relatively insoluble at pH > 5.5, in the concentrations required for the labeling reaction. We therefore radiolabeled the purified uncleaved recombinant fusion protein using the Bolton-Hunter reagent (Perkin Elmer Life Science, Boston, MA) following the manufacturer's specifications. Briefly, 250 μCi of Bolton-Hunter reagent were dried with N_2 _gas and chilled on ice and incubated with 1 mg recombinant fusion protein in 150 μl 0.1 M KH_2_PO_4_, pH 8.5 at 0°C for 1 h and then at 4°C overnight. The reaction was terminated by adding 100 μl of 1% glycine followed by 100 μl of 0.04% phenol red, and finally 100 μl of 0.01% gelatin, all in 0.1 M KH_2_, PO_4_, pH 8.5 buffer. Then, iodinated fusion protein was separated from free ^125^I by chromatography on a 10 ml PD-10 column, (Amersham Biosciences, Uppsala, Sweden) equilibrated in 0.1 M KH_2_PO_4_, pH 8.5. Fractions containing fusion protein were pooled, dialyzed against 100 volumes of 50 mM Tris, 150 mM NaCl, ph 7.4, (TBS) with 2.5 mM CaCl_2 _overnight, and cleaved with thrombin at a ratio of 1:250 (wt/wt) for 2 h at room temperature (25°C). [^125^I]mactinin was separated from GST by reverse phase C-4 HPLC as previously described [2].

### Specificity of mactinin binding to HL-60 cells

HL-60 cells were incubated in RPMI medium with or without 5 μM unlabeled (cold) mactinin for 30 min at room temp. Then 100 nM [^125^I]mactinin was added and the cells incubated at 37°C for 2 h. The cells were washed, lysed, clarified by centrifugation, the supernatant resolved by 12% SDS-PAGE under non-reducing conditions. [^125^I]mactinin was detected using autoradiography using Kodak BioMax MS film at -70°C.

### Labeling of HL-60 cell surface proteins with biotin

Since mactinin promotes the maturation of HL-60 cells as well as monocytes [5], and HL-60 cells are readily available as suspension culture cells in unlimited numbers with potentially several days of *in vitro *viability, they were used instead of monocytes for the biochemical assays. HL-60 cells (120 × 10^6^) were washed two times with PBS and then diluted to 2 × 10^6 ^cells/ml in PBS. NHS-LC-biotin (Pierce, Rockford, IL) was dissolved in DMSO and added to HL-60 cells at a concentration of 0.1 mg/ml of cells [46]. Samples were incubated at 4°C for 15 min and then washed three times with PBS + 50 mM NH_4_Cl to remove unbound biotin.

### Cross-linking of mactinin bound to HL-60 cells

One hundred twenty million biotin-labeled HL-60 cells were incubated with 100 nM [^125^I]mactinin for 1 h at 4°C with occasional mixing. Cells were then washed and resuspended in PBS. In some tubes, a membrane permeable and thio-cleavable cross-linker, 1 mM dithiobis [succinimidyl-proprionate] (DSP, Pierce) was added for 1 h at 4°C. In other tubes, the membrane-impermeable and non-cleavable cross-linker 1 mM bis(sulfosuccinimidyl)suberate (BS^3^, Pierce) was added. The cross-linking reaction was quenched with 20 mM Tris, pH 7.5 for 15 min at room temperature, the cells washed again with PBS and lysed in 500 μl of 50 mM Tris, pH 7.8, 5 mM CaCl_2_, 1% Triton, 2 mM PMSF, 10 μM pepstatin A. The lysate was centrifuged at 12,000 RPM for 10 min at 4°C, and the supernatant was further analyzed as described below.

### SDS-PAGE

Supernatant samples were concentrated using a speed-vac, mixed with an equal volume of gel loading buffer and the proteins resolved by 6–20% gradient SDS-PAGE. After electrophoresis, the gel was cut, and some lanes were dried and exposed to Kodak BioMax MS film at -70°C for detection of [^125^I]mactinin. From companion lanes, gel slices containing lysed HL-60 cells cross-linked to [^125^I]mactinin were electroeluted, dialyzed against TBS, concentrated and reduced with DTT, then run on a 5 ml column of recombinant mactinin covalently bound to an Affi-Gel 15 matrix. The material was loaded on the column, washed with 100 ml TBS, and eluted with 0.1 M sodium citrate, 0.3 M NaCl, pH 3.0. Two ml fractions were collected, neutralized with 2 M Tris, pooled and dialyzed against 10 mM Tris, pH 7.5 at 4°C. The eluate was concentrated and proteins resolved by 6–20% gradient SDS-PAGE under reducing conditions. After electrophoresis, the gel was transferred to an Immuno-Blot PVDF membrane (Bio-Rad). The membrane was blocked with 5% BSA-TBS, 0.05% Tween-20 for 1 h at room temperature with rocking, followed by incubation with 40 pg/ml streptavidin-horseradish peroxidase (Pierce) in 5% BSA-TBS, Tween for 1 h at room temperature with gentle rocking. The membrane was washed 5 times in TBS-Tween, and biotin-labeled proteins detected by SuperSignal West Femto enhanced chemiluminescence (ECL) substrate (Pierce) using the manufacturer's directions.

### Mass spectrometry

SDS-PAGE gel slices corresponding to the biotin-labeled proteins from the reduced high molecular weight complex were analyzed using a Waters Q-Tof mass spectrometer at the Keck Foundation Biotechnology Resource Laboratory (New Haven, CT). Trypsin digestion was carried out on the SDS-PAGE gel slices as previously described [5]. The mass spectrometer data were searched using the automated Mascot algorithm.

### Western immunoblotting

To further confirm the identity of the proteins in the mactinin-binding complex identified by mass spectrometry, biotin-labeled mactinin-binding proteins on the PVDF membrane were washed 5 times with TBS-Tween to remove the streptavidin-horseradish peroxidase and ECL substrate. The membrane was then treated with antiserum to Hsp90 (BD Bioscience), followed by alkaline phosphatase-conjugated secondary antibody (Sigma). Immunoreactive protein bands were detected by alkaline phosphatase reaction using 5-bromo-4-chloro-3-indoyl phosphate/nitroblue tetrazolium.

### Treatment of monocytes with Hsp90 inhibitors

The Hsp90 inhibitors geldanamycin, 17-AAG and 17-DMAG [31] (InvivoGen, San Diego, CA) were each dissolved in DMSO to a stock solution of 2 mmol/L. They were then diluted in RPMI media supplemented with 100 units/ml penicillin and 100 μg/ml streptomycin. After treatment, monocytes were incubated with 0.2% trypan blue and assayed for viability using light microscopy. The percentage of viable cells, which exclude trypan blue, was > 95% in all cultures.

### Chemotaxis assay

Cell migration was assessed, as previously described [4], in a 48-well micro chemotaxis chamber (NeuroProbe, Gaithersburg, MD). One nM mactinin or 1 nM formyl-methionyl-leucyl-phenylalanine (fmlp) was placed in the lower compartment. Monocytes (30–35,000) were treated for 30 min at 37°C with various Hsp90 inhibitors and then placed in the upper compartment of the well. The two compartments were separated by a 5-μm pore size PVP-free polycarbonate filter. The chamber was incubated for 90 min at 37°C in 5% CO_2_. At the end of the incubation period, the filter was removed, fixed, and stained with a Hema 3 stain set (Fisher, Pittsburgh, PA). The number of cells that migrated through the membrane pore in three high-power fields (×400) was counted by light microscopy. Three chamber membranes were counted for each treatment. The number of cells in the field of vision was multiplied to reflect the total cell number on the 8 mm^2 ^membrane.

### Statistical analysis

One-way analysis of variance (ANOVA) with Dunnett's multiple comparison post test correction was used for multiple comparisons. Student's *t*-test was used for single comparisons.

## Competing interests

The authors declare that they have no competing interests.

## Authors' contributions

SDL, RTP, KG, TRO and PG designed the research, analyzed data, and wrote the manuscript.

APM and TH designed and performed research, and analyzed data. All the authors read and approved the final manuscript.
